# Health Literacy in Oculofacial Plastic Surgery: A Literature Review

**DOI:** 10.7759/cureus.41518

**Published:** 2023-07-07

**Authors:** Narmien Murdock, Alexander Missner, Viraj Mehta

**Affiliations:** 1 Ophthalmology, MedStar Georgetown University Hospital, Washington, DC, USA; 2 Ophthalmology, Georgetown University School of Medicine, Washington, DC, USA

**Keywords:** patient education, readability, plastic surgery, ophthalmology, oculofacial, oculoplastic, health literacy

## Abstract

Patient satisfaction following oculofacial cosmetic procedures depends on preoperative expectations, which may be influenced by online material. Patients with poor health literacy are particularly vulnerable to misinformation and low-quality resources. However, few studies have evaluated the quality of online information on common oculofacial plastic surgeries and procedures. This study aimed to review the literature on the readability and quality of online material related to oculofacial plastic surgery. We conducted a systematic search of the PubMed/MEDLINE database and included 10 studies in our review. Among the readability scores reported in these studies, the lowest was 10, representing a tenth-grade reading level. Furthermore, the online materials were often rated as "poor" quality based on multiple grading scales. Our systematic review of the literature demonstrates that online materials covering common oculofacial plastic surgery procedures are consistently of poor quality and exceed the recommended readability level. Therefore, considering these online materials that influence patient expectations could enable oculofacial plastic surgeons to better tailor their preoperative counseling.

## Introduction and background

Over the past two decades, there has been a significant increase in facial plastic surgery. In 2020, 2.3 million cosmetic surgical procedures were performed in the United States [1]. Of those, eyelid surgery, specifically blepharoplasty, remains among the top five performed [1]. Most patients interested in aesthetic plastic surgery turn to the Internet and social media platforms for information, even prior to consultation with a surgeon [2-4]. While some online resources are reliable and serve to empower patients, intermixed among them are concepts that are inaccurate, outdated, or unrealistic [2,4,5]. Recognizing dependable resources and deciphering complex medical information can be challenging, particularly in patients with limited health literacy (HL) [6]. HL represents the ability to comprehend health information and utilize it to make appropriate medical decisions [7]. Its impact on patient morbidity and health outcomes has been extensively studied and extends to the surgical realm [8]. Limited HL has been shown to influence patient decision-making and satisfaction and affect final surgical results [8,9]. This is important to recognize since patients with various levels of HL rely on the Internet for health content and often carry the burden of evaluating its quality [10]. The utility of online health information depends in part on its readability or the ease with which it is read [11]. The American Medical Association (AMA) has recommended that patient education materials (PEMs) be written at or below the sixth-grade reading level [12]. PEMs above the ninth-grade level are considered difficult to read for an average adult [11,12]. Several studies in the current literature concluded that online plastic surgery PEMs are of low quality and written at unacceptably high reading levels [6,13,14]. Research investigating the quality and readability of PEMs specific to the field of oculofacial plastic surgery is sparse. Physicians can use these findings to assess and improve the effectiveness of the educational materials they provide, enhancing doctor-patient communication and shared decision-making. Addressing the readability and accessibility of these materials enables patients to comprehend complex medical information and promotes equitable access to information. This comprehensive literature review investigates the quality and readability of online material pertaining to oculofacial plastic surgery.

## Review

Methods 

A comprehensive literature search was conducted on the PubMed/MEDLINE database. No date restrictions were applied. A combination of terms related to online health information and oculofacial plastic surgery was used, such as “health literacy,” “online education,” and “oculofacial plastics.” Further strategies included searching subheadings and synonymous MeSH terms with the listed phrases in addition to including names for specific oculofacial procedures. Titles and abstracts of all studies were reviewed, followed by an assessment of full-text articles based on inclusion and exclusion eligibility. Studies that analyzed the readability and quality of online health information related to oculofacial plastic surgery were included. Exclusion criteria included the following: (1) reporting on plastic surgery procedures outside the realm of oculofacial plastics; (2) qualitative studies without quantitative analyses; (3) non-peer-reviewed studies; and (4) lack of availability in English. Data collected from eligible studies included objectives, the number of online items reviewed, average readability and/or quality scores, and conclusions.

Results

Database Review and Articles Included

Literature searches revealed a total of 40 studies, of which 10 were included (Table 1). The online material reviewed in these studies included brochures, web pages, and videos. Two studies evaluated PEMs covering a wide spectrum of pathology treated by oculofacial plastic surgery [11,15]. Online PEMs specific to blepharoplasty were evaluated in eight of the 10 studies, and, of these, two also assessed brow lift PEMs [14,16]. Seven out of the 10 studies reported on the quality of the online materials studied, and four articles focused on readability.

**Table 1:** Included studies assessing online materials in oculofacial plastic surgery. *Range provided due to various modalities used to assess readability. FRE: Flesch Reading Ease; FKGL: Flesch-Kincaid Grade Level; JAMA: Journal of the American Medical Association; EQIP: Ensuring Quality Information for Patients; SMOG: Simple Measure of Gobbledygook.

First Author & Year	Material Reviewed	Assessment Tool	Quality	Mean Reading Grade Level
Pakhchanian H et al. (2022) [15]	Online brochures	FRE, FKGL, SMOG	N/A	10–12*
Karataş ME and Karataş G (2022) [17]	Videos	DISCERN, JAMA	Fair (DISCERN), Poor (JAMA)	N/A
Om A et al. (2021) [18]	Videos	DISCERN	Poor	N/A
Alwani MM et al. (2020 [14]	Web pages	DISCERN	Fair	N/A
Gray MC et al. (2020) [16]	Videos	EQIP	High (“blepharoplasty”) Low (“forehead lift”)	N/A
Johnson AR et al. (2019) [19]	Web pages	SMOG	N/A	10.6
Ward B et al. (2019) [20]	Videos	DISCERN	Poor	N/A
Awal DH and Mills C (2018) [21]	Web pages	DISCERN, JAMA, FRE, FKGL	Poor (DISCERN), Poor (JAMA)	11
Huang G et al. (2015) [11]	Web pages	FRE, FKGL, SMOG	N/A	12–14*
Zaidi FH and Jones CA (2009) [22]	Web pages	JAMA	Poor	N/A

Analysis of Readability

Ensuring the readability of patient-directed health information is a key focus for the American Medical Association (AMA) and the National Institutes of Health (NIH) due to its impact on patient understanding. The current recommendation is that these resources should be written at a sixth-grade reading level, as it enhances accessibility and comprehension [12]. Various tools are available to assess the readability of PEMs, with the most commonly used ones being the Flesch Reading Ease (FRE) and Flesch-Kincaid Grade Level (FKGL) tests. The FRE score is calculated based on factors like syllable count and sentence length, with higher scores indicating better readability. A score between 60 and 70 corresponds to a reading level between eighth and ninth grade [15, 23]. On the other hand, the FKGL score also considers similar variables as FRE but determines the grade level, with higher scores indicating lower readability [23]. Among the articles reviewed, three studies utilized the FRE and FKGL tests to evaluate readability [11, 15, 21]. Three of the four articles in the current review that evaluated readability used FRE and FKGL tests (Table 1) [11,15,21]. Pakhchanian H et al. examined the readability of educational brochures from the American Society of Ophthalmic Plastic and Reconstructive Surgery (ASOPRS) covering various topics in oculofacial plastic surgery. The mean FRE score across all 18 brochures was 48, indicating a readability level below the recommended standard. The mean FKGL score was 11, highlighting the need for improved clarity and simplicity in these materials [15]. Huang G et al. also assessed ASOPRS PEMs, finding that the mean FRE and FKGL scores were 45 and 12, respectively, further emphasizing the need for enhanced readability [11]. Awal DH and Mills C reviewed 200 cosmetic facial surgery websites, 32 of which covered blepharoplasty specifically [21]. Among these 32 websites, the mean FRE was 51, and the mean FKGL was 11 [21]. Another tool that determines reading level based on syllable count is the Simple Measure of Gobbledygook (SMOG) test [24]. The total score is based on polysyllabic word count and is converted to a grade level, with higher scores indicating higher complexity and lower readability [24]. Three studies included in this review used the SMOG test [11,15,19]. The mean SMOG scores of ASOPRS online PEMs based on two of these studies were 13 and 14, corresponding to college-level readability [11,15]. Johnson AR et al. analyzed the readability of Spanish PEMs for the top five cosmetic surgeries using a modified SMOG scale [19]. Based on this scale, the mean reading grade level for websites discussing blepharoplasty was 10.6 [19]. While the reviewed studies predominantly focused on FRE, FKGL, and SMOG, it is essential to note that other validated assessments for evaluating the readability of PEMs exist (Table 2) [15, 25, 26].

**Table 2:** Summary of reading and analysis of quality scores.

Readability Score	Author	Description
DISCERN	Charnock D et al. (1999) [27]	The DISCERN tool is a 16-question tool that assesses the quality of patient-directed health information. The tool focuses on the following five domains: reliability, clarity, balance, overall quality, and patient involvement. Each question is scored on a scale of one to five, with a higher score indicating a higher quality of information. The total score ranges from 0 to 80, with a score of 68 or higher indicating high-quality information.
Flesch Reading Ease (FRE)	Flesch R (1948) [23]	The FRE score is a measure of how easy a text is to read. A score of 100 is the easiest to read, while a score of zero is the most difficult to read. FRE scores are calculated based on the average number of syllables per word and the average sentence length. An FRE score of 60-70 is considered to be at the 9th-grade level and, therefore, readable to the general population.
Flesch-Kincaid Grade Level (FKGL)	Flesch R (1948) [23]	The FKGL is a measure of the US grade level required to understand a text. A score of zero is equivalent to kindergarten, while a score of 12 is equivalent to the 12th grade. FKGL scores are calculated based on the FRE score and the number of words in the text. An FKGL score of eight or lower is considered to be easy to read for the general population.
JAMA Quality-Assessment Tool For Educational Webpages (JAMA)	Silberg WM (1999) [28]	The JAMA quality-assessment tool is a four-point quality-assessment tool for educational web pages. The four areas emphasized in this tool include the following: clear declaration of authorship and affiliation, the presence of references for all information, disclosure of conflicts of interest, and how current or updated the presented information is.
Ensuring Quality Information For Patients (EQIP)	Moult B et al. (2004) [29]	The EQIP is a 20-question quality-assessment tool for written patient-directed health information (PEM). EQIP focuses on the completeness, appearance, understandability, and usefulness of PEM.
Simple Measure Of Gobbledygook (SMOG)	Mclaughlin HG (1969) [24]	The SMOG is a measure of how difficult a text is to understand. A score of 0 is the easiest to understand, while a score of 4 is the most difficult to understand. SMOG scores are calculated based on the number of polysyllabic words in a text and the average sentence length. A SMOG score of 2-3 is considered to be easy to read for the general population, and a SMOG of 15 being difficult to read.

Analysis of Quality

Multiple tools have been developed with the purpose of measuring the quality of PEMs. The DISCERN score is a validated and commonly used tool that is based on the reliability, specificity, and overall quality of the provided information [27]. The assessment consists of 16 questions, each rated on a scale from one to five, with 80 being the highest possible quality score. While the DISCERN handbook does not include cutoff points for score interpretation, the following score grading has been proposed by Alwani MM et al. and applied to the DISCERN scores reported in this review: "very poor" (under 28), "poor" (29-41), "fair" (42-54), "good" (55-67), "excellent" (68-80) [14]. Of the seven studies evaluating online health information quality, five used the DISCERN tool (Table 1) [14,17,18,20,21]. Om A et al. analyzed the quality of aesthetic surgery procedure videos on the social media platform, TikTok [18]. Among the 50 "blepharoplasty" videos reviewed, the mean DISCERN score per question was 1.44 out of five, giving a total score of approximately 23 [18]. Alwani MM et al. reported the highest quality PEMs among the included studies, with mean DISCERN scores of 47.1 and 44.6 for blepharoplasty and brow lift PEMs, respectively [14]. The Journal of the American Medical Association (JAMA) published a four-point quality-assessment tool for educational webpages [28]. The four areas emphasized in this tool include the following: (1) a clear declaration of authorship and affiliation; (2) the presence of references for all information; (3) disclosure of conflicts of interest; and (4) how current or updated the presented information is [28]. Three of the studies included here utilized the JAMA tool [17,21,22]. Zaidi FH and Jones CA analyzed 101 websites reporting information on blepharoplasty, of which 81% had a total quality score of zero to one out of four [22]. Karataş ME and Karataş G analyzed 186 videos reviewing blepharoplasty and reported a mean JAMA score of 1.39 [17]. Awal DH and Mills C reported that the mean JAMA score of 32 websites on blepharoplasty was 0.66 [21]. A more recently developed quality-assessment tool is the Ensuring Quality Information for Patients (EQIP), which consists of 20 questions that focus on the completeness, appearance, understandability, and usefulness of written PEMs [29]. One study included here used a 27-point modified EQIP tool to assess the quality of 523 aesthetic videos [16]. Scores above 14.3, corresponding to the 75th percentile, were labeled "high quality." The mean scores for the "blepharoplasty" and "eyelid surgery" videos were 14.36 [16]. Videos with information on "forehead lift" had a lower mean score of around 12 (Table 3) [16].

**Table 3:** Average reading and quality scores.

Tool	Author	Average Scores by Study	Calculated Average for Each Score	Interpretation: Readability or Quality
Flesch Reading Ease (FRE)	Pakchanian H et al. (2022) [15]	48	48	Moderately difficult to read
	Huang G et al. (2014) [11]	45		
	Awal DH and Mills C (2018) [21]	51		
Flesch-Kincaid Grade Level (FKGL)	Pakchanian H et al. (2022) [15]	11	11.33	Beyond high-school readability
	Huang G et al. (2014) [11]	12		
	Awal DH and Mills C (2018) [21]	11		
Simple Measure of Gobbledygook (SMOG)	Pakchanian H et al. (2022) [15]	14	13.5	College-level readability
	Huang G et al. (2014) [11]	13		
	Johnson AR et al. (2019) [19]	10.6 (Modified SMOG)	10.6	
DISCERN Score	Om A et al. (2021) [18]	23	38.48	Poor-to-fair quality
	Alwani MM et al. (2020) [14]	47.1 (blepharoplasty) & 44.6 (brow lift)		
	Karataş ME and Karataş G (2022) [17]	49.85 (physician uploaded PEM) 27.85 (non-physician uploaded PEM)		
Journal of the American Medical Association (JAMA)	Zaidi FH and Jones CA (2009) [22]	0-1	0.77	Low quality
	Karataş ME and Karataş G (2022) [17]	1.70 (physician uploaded PEM) 0.23 (non-physician uploaded PEM)		
	Awal DH and Mills C (2018) [21]	0.66		
Ensuring Quality Information for Patients (EQIP)	Moult B et al. (2004) [29]	14.36	14.36	High quality

Karataş ME and Karataş G reported that the quality of videos uploaded by physicians was of higher quality compared to those uploaded by non-physicians [17]. The mean DISCERN scores for physician and non-physician videos were 49.85 and 27.38, respectively (P < 0.001). The mean JAMA scores for physician and non-physician videos were 1.70 and 0.23, respectively (P < 0.001) [17].

Discussion

As the demand for cosmetic surgery increases, patient HL remains important due to its impact on surgical decision-making and outcomes [8]. Studies have demonstrated that nearly 50% of patients may be affected by marginal or low HL, reading below an 8th-grade level [30]. These patients are also less likely to ask questions during their medical visits due to feelings of shame [31,32]. The accessibility of health information online allows patients to turn to the Internet for answers and advice. Since recognizing reliable resources and accurate information can be challenging, healthcare practitioners should ensure patient access to high-quality information that is easy to read [33]. The issue of inadequate PEM quality and readability has been reported in multiple surgical specialties, including plastic surgery [13,34-37]. Prior studies have demonstrated that plastic surgery PEMs, even when provided by surgical societies and academic institutions, are of low quality [14,38]. Herein, the authors report the first literature review analyzing the status of online PEM quality and readability within the field of oculofacial plastic surgery. In current literature, the few studies evaluating the readability of online oculofacial plastic surgery material reported that PEMs were at a 10th-grade reading level or higher, which is well above the recommended guidelines of the AMA and NIH [7,11,12,15]. These findings suggest that oculofacial plastic surgery PEMs are written at a level that is difficult for the average adult patient to understand. Healthcare agencies, such as the NIH, have published recommendations for improving the readability of PEMs. These include writing medical information in clear and concise "plain language" and using simple, direct sentences and common, everyday words when possible [7,39]. Additionally, using visual aids and illustrations is encouraged and can be very effective. Social media platforms, such as TikTok and YouTube, allow the dissemination of educational videos, which can be especially useful for individuals with low literacy [40,41]. Another important intervention that improves PEM readability is incorporating culturally sensitive resources in multiple languages [39]. Patients that are particularly vulnerable to low HL are the elderly and non-native English speakers [42]. In this review, English and Spanish were the only languages included in readability assessments of oculofacial plastic surgery PEMs. Johnson AR et al. reported that blepharoplasty educational material written in Spanish was of low readability and cultural sensitivity [19]. This literature review demonstrates that the quality of online oculofacial plastic surgery information is inadequate for patient education. All included studies that used the DISCERN instrument reported a score of below 55, which is considered the lower limit for "good quality" PEMs [14,43]. Only Gray MC et al. reported that videos with information on blepharoplasty were of "high quality" [16]. However, this was based on a mean EQIP score of 14.36, with 14.3 being the cutoff between high- and low-quality information [16]. Interestingly, certain studies found that the quality of content produced by physicians was higher than that produced by non-physicians [16,17,20]. Ward B et al. also demonstrated that YouTube videos involving specialty-related, board-certified physicians had the highest patient utility [20]. This highlights the tendency for patients to search for and view content presented by credentialed and reputable providers. Therefore, online PEMs should include a qualified author or reference with specific credentials [22,44]. Oculofacial plastic surgeons should be aware of the impact online PEMs can have on patient satisfaction, adherence to post-operative instructions, and surgical outcomes. Providers have a responsibility to create and/or share reliable, patient-oriented content that is comprehensible to individuals of various HL levels. This requires knowledge of the characteristics that define high-quality and readable PEMs. For example, patient-oriented material discussing blepharoplasty should include the aims and benefits of the procedure, its potential risks and complications, alternative interventions, and clear sources from which information was gathered [14,38]. Many tools, such as those presented in this study, exist to help determine the objective quality and readability of online PEMs. Future research endeavors can shift the focus from the quality and readability of current PEMs to the assessment of HL in oculofacial plastic surgery patients. Additionally, future studies can investigate which online platforms are most utilized by cosmetic surgery patients. Although research on HL in oculofacial plastic surgery is sparse, multiple studies propose solutions to improve the quality of patient-surgeon communication and address HL in plastic surgery [13,45,46]. There should be an effort to incorporate tools that have proven effective in other surgical specialties into the field of oculofacial plastic surgery [45,47-49].

## Conclusions

This comprehensive literature review suggests that online PEMs discussing topics and procedures in oculofacial plastic surgery are of low quality and readability. This increases the risk of lower patient satisfaction and poor surgical outcomes, particularly among patients with limited HL. Some studies have found that PEMs created by physicians tend to be of higher quality and have more patient views. Oculofacial plastic surgeons have a responsibility to create and/or share online resources that are credible and comprehensible to patients of various backgrounds and HL levels. These efforts can improve the surgical decision-making process and overall patient health outcomes.
